# Age-related abnormalities of thalamic shape and dynamic functional connectivity after three hours of sleep restriction

**DOI:** 10.7717/peerj.10751

**Published:** 2021-01-26

**Authors:** Zhiliang Long, Jia Zhao, Danni Chen, Xu Lei

**Affiliations:** 1 Sleep and NeuroImaging Center, Faculty of Psychology, University of the Southwest, Chongqing, China; 2 Key Laboratory of Cognition and Personality (Southwest University), Ministry of Education, Chongqing, China

**Keywords:** Sleep loss, Aging, Thalamus, Subcortical shape, Functional connectivity variability

## Abstract

**Background:**

Previous neuroimaging studies have detected abnormal activation and intrinsic functional connectivity of the thalamus after total sleep deprivation. However, very few studies have investigated age-related changes in the dynamic functional connectivity of the thalamus and the abnormalities in the thalamic shape following partial sleep deprivation.

**Methods:**

Fifty-five participants consisting of 23 old adults (mean age: 68.8 years) and 32 young adults (mean age: 23.5 years) were included in current study. A vertex-based shape analysis and a dynamic functional connectivity analysis were used to evaluate the age-dependent structural and functional abnormalities after three hours of sleep restriction.

**Results:**

Shape analysis revealed the significant main effect of deprivation with local atrophy in the left thalamus. In addition, we observed a significant age deprivation interaction effect with reduced variability of functional connectivity between the left thalamus and the left superior parietal cortex following sleep restriction. This reduction was found only in young adults. Moreover, a significantly negative linear correlation was observed between the insomnia severity index and the changes of variability (post-deprivation minus pre-deprivation) in the functional connectivity of the left thalamus with the left superior parietal cortex.

**Conclusions:**

The results indicated that three hours of sleep restriction could affect both the thalamic structure and its functional dynamics. They also highlighted the role of age in studies of sleep deprivation.

## Introduction

Sleep loss is a common and serious issue that has exerted a negative effect on health nowadays. Individuals suffering from sleep loss have a low quality of life and a high likelihood of experiencing accidents. Insufficient sleep has been associated with the impairment of cognition and emotion (Lim & Dinges, 2010). The sleep–wake cycle has been observed to be disrupted in psychiatric and neurological disorders (Videnovic et al., 2014). Thus, elucidating the mechanisms underlying sleep deprivation is an important goal in basic and clinical neuroscience.

Research has suggested that insufficient sleep is involved in the dysfunction of the thalamus. Neuroimaging task studies have reported that the abnormal activity of the thalamus after total sleep deprivation is associated with impaired sustained attention (Chee et al., 2010; Chee et al., 2008). Resting-state functional magnetic resonance imaging (fMRI) technique, which captures intrinsic brain activity, has caused lots of attention (Liu et al., 2013; Liu et al., 2012). For example, studies on resting-state fMRI have observed alterations in the amplitude of low-frequency fluctuations (ALFF) of the thalamus (Chen, Qi & Zheng, 2018) and decreased thalamic functional connectivity (FC) with cortical brain areas, such as the anterior and posterior cingulate cortex (Yeo, Tandi & Chee, 2015) and the superior and medial prefrontal cortex (Shao et al., 2013). Moreover, the FC between the thalamus and the default mode network can predict worse working memory performance following sleep deprivation (Chengyang et al., 2017; Lei et al., 2015). However, these studies are all based on the assumption that the resting-state FC is stationary during scanning, thus ignoring the dynamic features of FC on a smaller time scale (Allen et al., 2014; Preti, Bolton & Ville, 2017). Sleep deprivation studies have shown altered dwell time and transit between FC states using whole brain dynamic FC analysis (Li et al., 2020; Xu et al., 2018). Hence, the effects of sleep deprivation on the dynamics of thalamic FC remain unclear.

Additionally, substantial evidence has suggested that individuals with different age cohorts have different sensitivities to losing sleep. For example, old people tolerate sleep deprivation better than young people (Duffy et al., 2009). The decrements of bad cognitive performance after insufficient sleep are higher in young adults than in old adults (Richards et al., 2017). A verbal encoding task reported an increased activation of the anterior parahippocampus following sleep deprivation in old adults; however, this activation decreases in young adults (Jonelis et al., 2012). Studies on resting-state fMRI also have reported altered FC of insula after sleep deprivation (Long & Cheng, 2019) and the association of FC of the medial temporal lobe with sleep quality only in young people (Liu et al., 2018). Nevertheless, research has not clearly determined whether age could affect the alteration of thalamic functional dynamics caused by sleep deprivation.

The brain structure has been thought to constrain the brain function significantly (Honey et al., 2009). Thus, the structural substrate of brain dysfunction should be studied to gain full understanding of the mechanism underlying sleep deprivation. Structural MRI studies have already observed gray matter volume loss in the thalamus following sleep deprivation by using voxel-based morphometry (VBM) (Liu et al., 2014; Long, Cheng & Lei, 2020). Currently, a vertex-based shape analysis has emerged to investigate the local atrophy in subcortical structures (Patenaude et al., 2011). This method overcomes some limitations of VBM, such as reliance on the extent of arbitrary smoothing (Patenaude et al., 2011). Thus, researchers believe that local subcortical abnormalities are detected more precisely in the shape analysis than in VBM (Kim et al., 2013). Indeed, some studies have demonstrated subcortical structural abnormalities using shape analysis, which were not detected in VBM (Kim et al., 2013; Štěpán Buksakowska et al., 2014). Shape analysis has revealed alteration of subcortical regions in sleep disorders (Macey et al., 2018; Rahayel et al., 2018). However, whether thalamic shape is altered following sleep deprivation remains unclear.

Previous studies that focused on partial sleep deprivation detected deprived alteration of intrinsic FC and activation of thalamus (Nilsonne et al., 2017; Tamm et al., 2017). However, research has not comprehensively explored whether the thalamic shape and dynamics are altered after partial sleep deprivation. By combining vertex-based shape analysis and resting-state dynamic FC analysis, the current study investigates age-related local structural abnormalities of the thalamus and its dynamic FC changes after three hours of sleep restriction. On the basis of previous findings, we hypothesize that thalamic atrophy and age-related alteration of FC variability are observed after three hours of sleep restriction.

## Materials and Methods

### Study design and participants

The dataset was obtained from the Sleepy Brain Project (https://openneuro.org/datasets/ds000201). Participants were recruited by poster and newspaper advertising. The criteria of inclusion were: (1) those required to undergo fMRI procedures, e.g., no ferromagnetic items in the body, not claustrophobic, and not pregnant; (2) those who have no current or past self-reported psychiatric or neurological illness; (3) those who do not have hypertension or diabetes; (4) those who do not use psychoactive or immune-modulating drugs; (5) those who do not consume nicotine every day. A total of 86 participants, including forty-seven young adults (20–30 years old) and thirty-nine old adults (65–75 years old), were included.

The detailed study design can be found in previous studies (Long, Cheng & Lei, 2020; Nilsonne et al., 2017; Tamm et al., 2017) and online service (https://www.protocols.io/view/three-hours-sleep-restriction-protocol-bqdrms56). The project was preregistered at clinicaltrials.gov (https://clinicaltrials.gov/ct2/show/NCT02000076). This study was approved by the Regional Ethics Review Board of Stockholm (2012/1870–32). All participants provided written informed consent before participating in this work, and all experiments were performed in accordance with the Declaration of Helsinki and applicable local regulations.

### Scan acquisition

The MRI data of the Sleepy Brain Project were acquired using a General Electric Discovery 3T MRI scanner. T1 structural images were scanned using a sagittal brain volume (BRAVO) sequence with the following values: repetition time (TR) = 6.4 s, echo time (TE) = 2.8 s, flip angle = 11°, 1 × 0.47 × 0.47 mm^3^ voxel size, 24-cm field of view, 1-mm slice thickness, and 3.58 min of acquisition time. Meanwhile, echo-planar images were acquired using the following settings: flip angle = 75°, TE = 30 ms, TR = 2.5 s, field of view = 28.8 cm, slice thickness = 3 mm, 2.25 × 2.25 × 3 mm^3^ voxel size, 49 slices, 192 scans, and a total duration of 8 min (Nilsonne et al., 2017; Tamm et al., 2017).

### Thalamic shape analysis

Shape analysis was performed using FIRST, which was implemented in the FSL toolbox, in a mode of operation that aims to assess group differences on a per-vertex basis. First, the T1 image of each subject was used to segment all the subcortical structures, including the thalamus. The vertex locations from each subject were then projected onto the surface normal of the group-average shape. Thus, the projection values were obtained. A positive value indicated that the anatomical point was outside the surface. In contrast, a negative value denoted that the point was inside the surface. To control for inter-individual head size differences, the meshes were reconstructed in the Montreal Neurological Institute (MNI) space.

The effect of age on the shape changes after sleep restriction was investigated by employing two-way mixed analysis of variance (ANOVA) (between-subject factor: age, two levels; within-subject factor: deprivation, two levels) in the “randomize” procedure. The ISI score was included as covariate. A family-wise error correction method with *p* < 0.05 (voxel *p* < 0.01) was used to correct multiple comparisons at the cluster level. Once a significant deprivation-related effect was observed, clusters from the statistical maps were saved as regions of interests (ROIs), which were used for post-hoc analysis. For the age × deprivation interaction effect, we separately investigated whether sleep-deprived young adults and old adults had atrophy in the thalamus. For the main effect of deprivation, we explored whether the projection values increased or decreased following the sleep restriction. The Bonferroni method with *p* < 0.05/6 was used for correcting the multiple tests.

Pearson correlation analysis was exploringly conducted to determine linear correlations between changes (post-deprivation vs. pre-deprivation) in the projection value of the ROIs and participant measures, including ESS, ISI, KSQ sleep quality index, snoring symptom index, and changes of the PANAS score. The statistical level of *p* < 0.05 was considered significant.

### Preprocessing of resting state scans

The preprocessing of the resting-state MRI data was conducted using the SPM12 software toolbox. The first 10 points for time were discarded because of the adaptation of participants to the scanning environment and the magnetization stabilization. The images were then corrected for the time-delay between slices and the motion movement between volumes. Participants with *x*, *y*, or *z* directions larger than 1.5 mm or rotation around each axis larger than 1.5° were excluded. Next, normalization was performed on the resulting images by using a unified segmentation of anatomical images, which were resampled into a voxel size of 3 × 3 × 3 mm^3^. A multiple regression model was used to remove the effect of the covariance of no interests, including 24 motion parameters, white matter signals, and cerebrospinal fluid signals. The resulting images were finally linearly detrended and filtered at the range of 0.01 Hz to 0.08 Hz.

### Seed-based dynamic FC analysis

The bilateral thalamus were obtained from the automated anatomical labeling atlas (Tzourio-Mazoyer et al., 2002), which was considered the seed ROI. Dynamic FC analysis was performed using the Temporal Dynamic Analysis toolkit based on the DPABI software (Yan et al., 2016). The window length is an important parameter in the computation of resting-state dynamics. In the current study, a moderate-length sliding window of 30 TR (75 s) was used (Deng et al., 2016; Xu et al., 2018). Research has suggested that the window length influences the accuracy of dynamic FC estimation. To ensure the consistency of our finding, we also analyzed the dynamic FC with window lengths of 20 TR and 40 TR. The window was moved with a step size of 1 TR (2.5 s).

For each ROI, a seed-based FC map was computed within each sliding window between the averaged time course of the seed ROI and the time series of left voxels by using Pearson’s correlation analysis. A set of sliding window correlation maps was obtained for each subject. These correlation maps further underwent r-to-z transformation to improve the normality of the distribution of correlation. The FC variability, which was referred to as FC flexibility (Song et al., 2019), was calculated for each subject. The value *v*(*i*) of the FC variability was the standard deviation of the connection strength between voxel *i* and the thalamus (seed ROI) across temporal windows. The analysis of the effect of age on the thalamic FC variability changes was performed using two-way mixed ANOVA with ISI score included as covariate. Multiple comparison correction was employed using the Gaussian Random Field method with cluster *p* < 0.05 and voxel *p* < 0.01. The post-hoc analysis and correlation analysis were the same as the procedures mentioned above.

## Results

**Figure 1:**
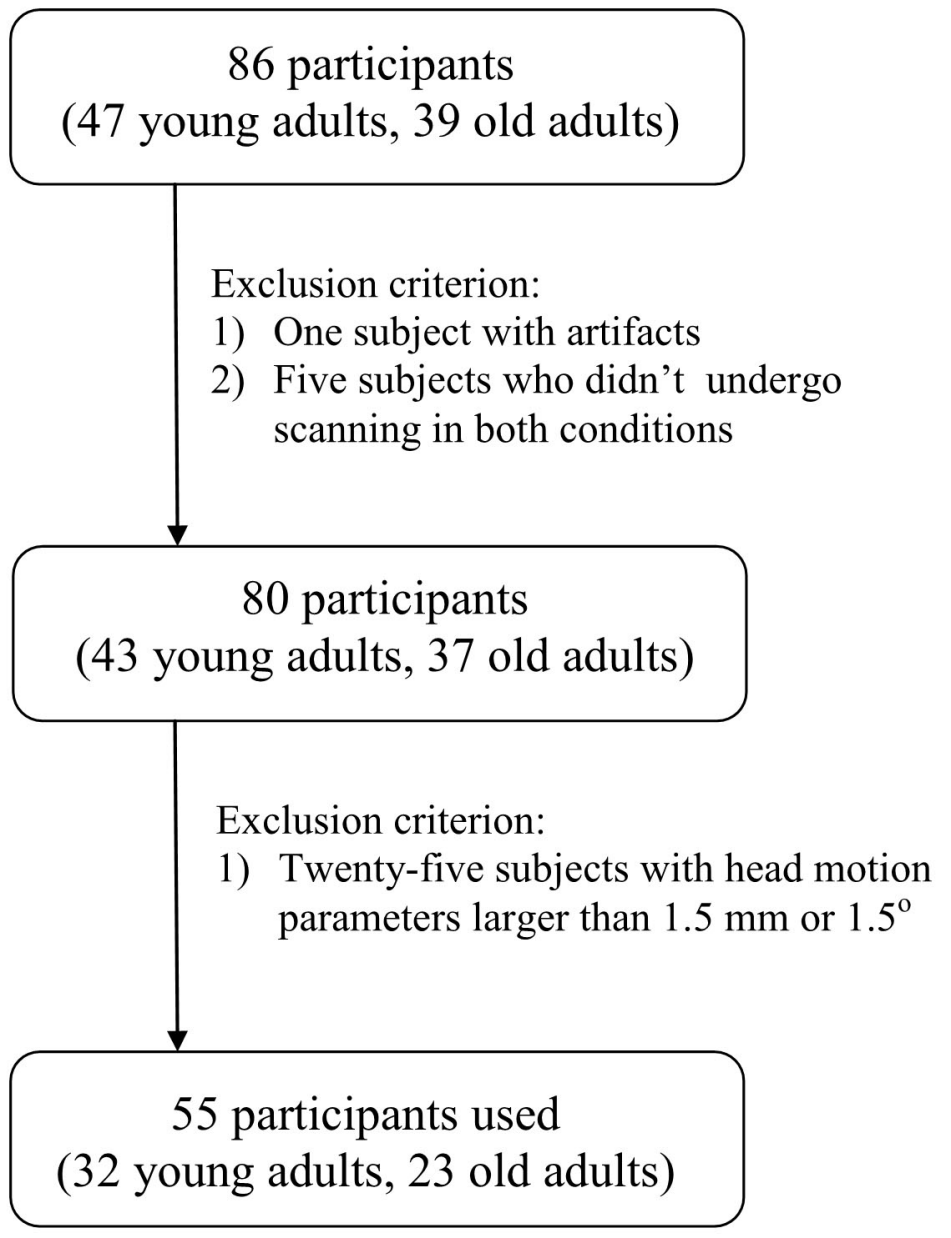
Flowchart. Flowchart of subject screening.

The subject screening procedure can be seen in Fig. 1. Five subjects were excluded because they did not undergo T1 image scanning before or after sleep restriction. One subject was excluded due to artifacts. Finally, 55 participants remained (23 old adults, 32 young adults).

The sleep diaries revealed that young and old participants had normal sleep times of 8.46 ± 0.79 h and 8.34 ± 0.85 h, respectively. Ambulatory polysomnography data showed that the total sleep times in the sleep restriction condition were 2.97 ± 0.59 h for young adults and 2.52 ± 0.4 h for old adults. In the normal sleep condition, the total sleep times were 6.71 ± 1.48 h and 5.56 ± 1.34 h for young and old groups, respectively. The PANAS score was recorded in both conditions. So, mixed ANOVA (two factors: age and deprivation) was used to test difference in the PANAS scores. For a positive PANAS score, we only observed the significant main effect of deprivation (*F* = 5.36, *p* = 0.025) with a decreased PANAS positive score after sleep restriction. We did not find any significant effects for the PANAS negative score. No significant differences (*p* > 0.05) were observed in the gender, ESS, KSQ sleep quality index, and KSQ snoring symptom index between young adults and old adults. The old adults had a significantly (*p* < 0.05) lower ISI score than young adults (Table 1).

**Table 1:** Participant characteristics and sleep measures.

	Young (*n* = 32)	Old (*n* = 23)	*p*-value
Gender (female/male)	17/15	14/9	0.57^a^
Age (mean ± SD)	23.5 ± 2.42	68.8 ± 2.62	**<0.001**
ESS (mean ± SD)	7.16 ± 2.73	8.96 ± 5.35	0.11^b^
ISI (mean ± SD)	10.41 ± 2.11	8.87 ± 1.46	**0.004**^b^
KSQ sleep quality index (mean ± SD)	5.29 ± 0.47	5.18 ± 0.45	0.41^b^
KSQ snoring symtom index (mean ± SD)	5.84 ± 0.37	5.78 ± 0.52	0.61^b^

**Notes.**

a Chi-square test.

b Two-tailed two sample *t*-test.

Bold *p*-values indicate significant difference in sleep measures between young group and old group.

SD standard deviation ESS Epworth Sleepiness Scale ISI Insomnia Severity Index KSQ Karolinska Sleep Questionnaire

**Figure 2:**
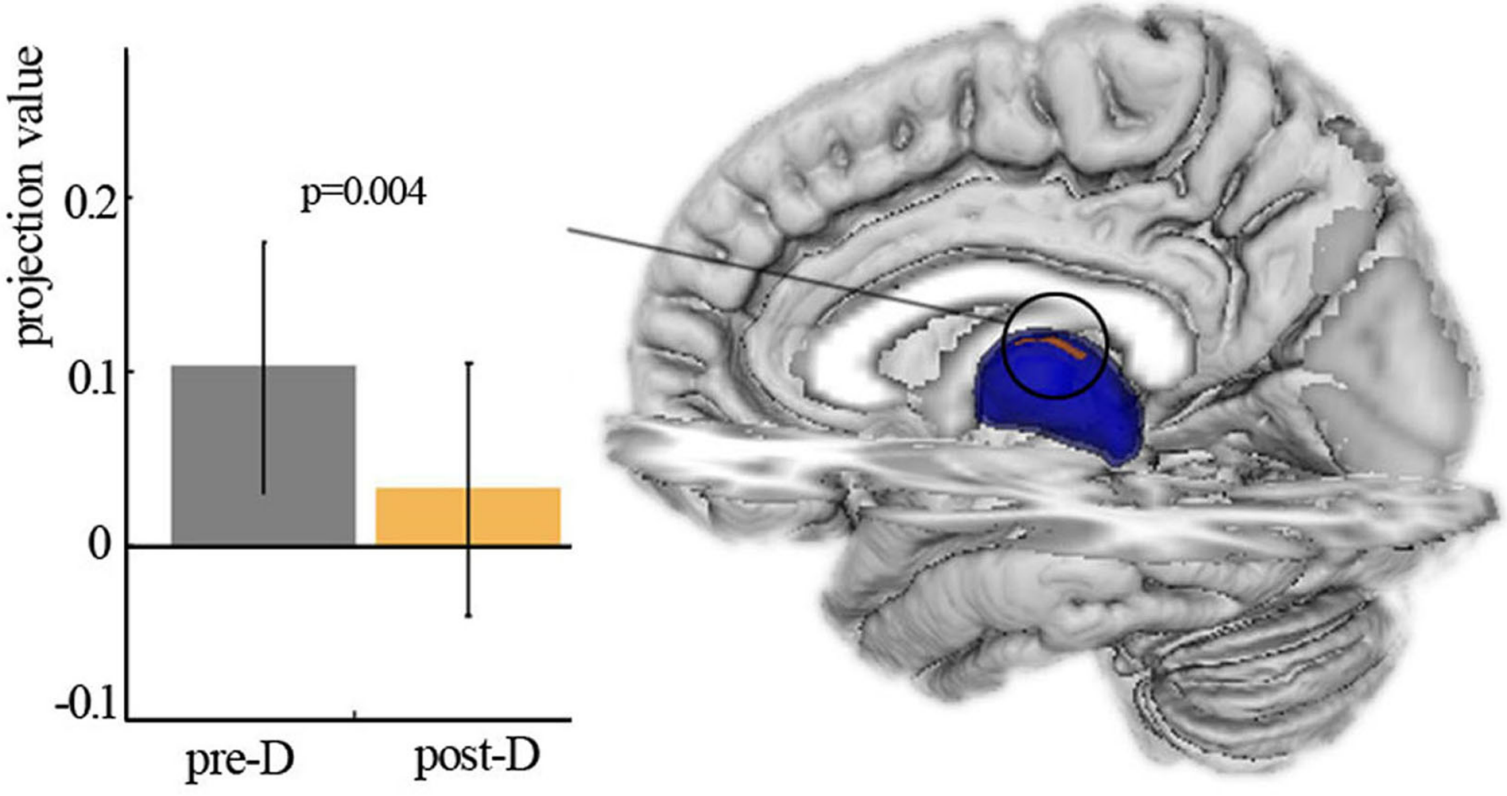
Shape analysis. Vertex-based shape analysis revealed significant main effect of deprivation with local atrophy in left thalamus. Pre-D, pre-deprivation; ost-D, post-deprivation.

Shape analysis revealed the significant main effect of deprivation with decreased projection value in the left thalamus after three hours of sleep restriction (Fig. 2). No significant main effect of deprivation was observed in the right thalamus, and no significant age × deprivation interaction effect was present in the bilateral thalamus. The changes of projection value were not correlated with participant measures.

**Figure 3:**
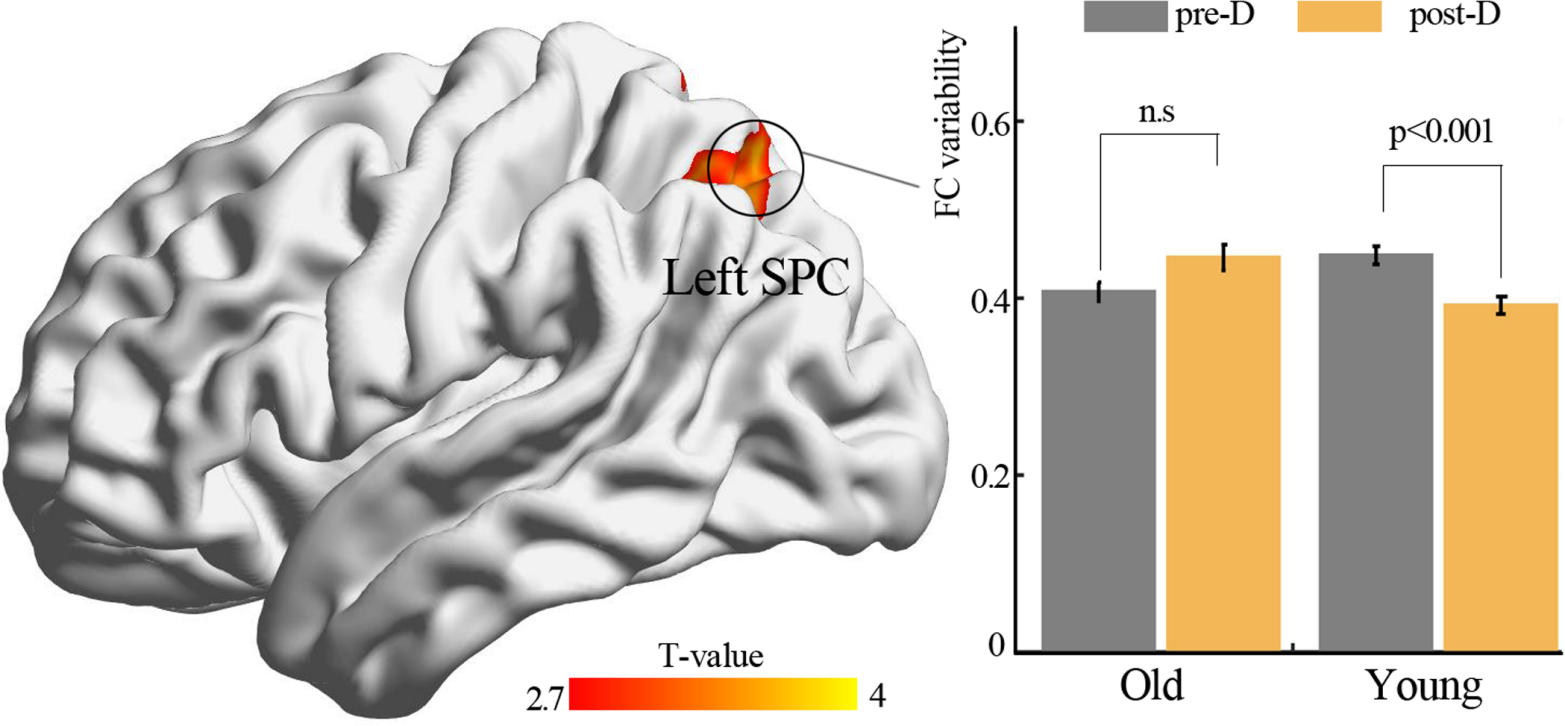
Functional connectivity variability analysis. Significant age × deprivation interaction effect with decreased functional connectivity variability of left thalamus (seed) after three hours of sleep restriction. SPC, superior parietal cortex; pre-D, pre-deprivation; post-D, post-deprivation; n.s., no significance.

**Figure 4:**
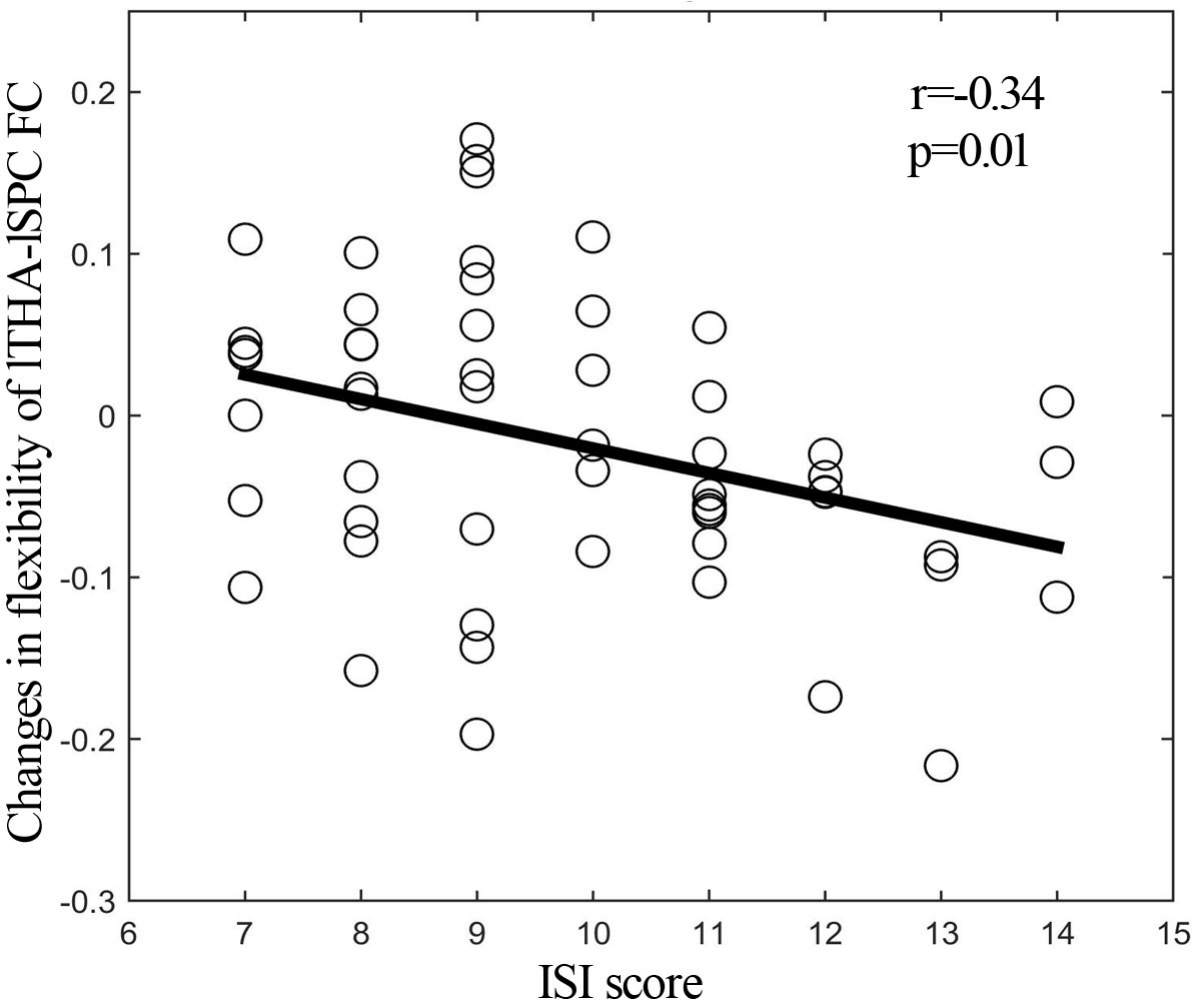
Correlation analysis. Significantly negative correlation between ISI score and changes (post-deprivation minus pre-deprivation) in functional connectivity variability of left thalamus and left SPC. ISI, Insomnia Severity Index; lTHA-lSPC FC, functional connectivity between left thalamus and left superior parietal cortex (SPC).

By using resting-state temporal dynamic FC analysis, we observed a significant age × deprivation interaction effect of the left thalamic FC variability. Post-hoc analysis showed that three hours of sleep restriction decreased the FC variability between the left thalamus and the left superior parietal cortex (SPC), which was observed only in young adults (Fig. 3, Table 2). The reduced variability of the left thalamic FC with the left SPC was still found when the window lengths of 20 TR and 40 TR were used (Figs. S1 and S2). We did not find significant main effect of deprivation. Additionally, a significantly negative linear correlation (*r* = −0.34, *p* = 0.01) was observed between the ISI score and the changes in variability (post-deprivation minus pre-deprivation) of the left thalamic FC with the left SPC (Fig. 4).

**Table 2:** Significant age × deprivation interaction effect of functional connectivity variability.

Brain areas	Voxel size	BA	Peak coordinate			Statistical value
			*x*	*y*	*z*	
*Seed: left thalamus*						
Left SPC	153	7	−27	−63	51	3.63

**Notes.**

SPC superior parietal cortex BA brodmann area

## Discussion

The current study investigated age-related local structural abnormalities of the thalamus and the changes of the thalamic FC variability after three hours of sleep restriction. We found significant atrophy in the left thalamus and reduced FC variability between the left thalamus and the left SPC in young adults. Additionally, the change of FC variability between the left thalamus and the left SPC was negatively correlated with the ISI score.

The thalamus represents a pivotal gating hub, which sends arousal signals from brainstem to cortical brain areas. Functional neuroimaging studies showed that impaired sustained attention after sleep deprivation was involved in abnormal thalamic activity. Such abnormal activity of the thalamus resulted in altered ascending arousal signals and then affected the cortical attentional networks (Doran, Dongen & Dinges, 2001; Goel et al., 2009). However, the direction of the activity changes within the thalamus was not consistent. Some studies have reported greater sleep-deprived activity (Chee & Choo, 2004; Chee et al., 2010), whereas other studies have found diminished thalamic activity (Chee & Tan, 2010; Chee et al., 2008). These inconsistences may be explained in the context of different task performances (Chee & Tan, 2010; Goel et al., 2009; Thomas et al., 2000). Structural neuroimaging studies have further reported reduced gray matter volume of the thalamus after sleep deprivation (Liu et al., 2014; Long, Cheng & Lei, 2020), thus providing structural evidence for thalamic dysfunction. The thalamic atrophy observed in the current study is in line with previous findings, thus strongly suggesting sleep-deprived dysfunction of the thalamo-cortical connectivity.

In addition, we observed a significant age × deprivation interaction effect with reduced FC variability between the left thalamus and the left SPC after sleep restriction in young adults. A previous study did not report altered thalamo-parietal FC after sleep deprivation using stationary FC analysis (Shao et al., 2013). However, the reduction of the thalamo-parietal FC variability observed in the current study might provide new insight into understanding the mechanism of sleep deprivation. The result was consistent with a study reporting a decreased number of transitions between brain states, which suggested reduced cognitive flexibility after sleep deprivation (Teng et al., 2019). The SPC is one of key regions of the fronto-parietal network (FPN), which has the ability to adapt to a wide variety of tasks dynamically and flexibly. Damages to the FPN may lead to cognitive control dysfunction, such as impaired attention and working memory, which was observed in sleep-deprived individuals (Chee et al., 2010; Choo et al., 2005). The decreased variability of the thalamo-SPC FC following sleep restriction found in the current study might indicate reduced ability to switch between tasks. Moreover, we observed a negative correlation between the changes in the thalamo-SPC FC variability and the ISI score, thus indicating that more severe insomnia entails more reduction in the variability of thalamo-SPC FC following sleep deprivation. This finding suggested that insomnia might be associated with dysfunction of the SPC.

Interestingly, the sleep-deprived reduction of FC variability was not found in old adults. A behavioral study found that old adults had less sleep requirement during daytime and reduced nocturnal slow-wave sleep compared with young adults (Dijk et al., 2010). Thus, young people were more sensitive to sleep deprivation than old people (Duffy et al., 2009). In addition, Zitting and colleagues (Zitting et al., 2018) found that young adults were more vulnerable to chronic sleep deficiency and recurrent circadian disruption and especially had more attentional failures than older adults. The higher ISI score in young adults observed in the current study provided direct evidence of this supposition. These findings were in accordance with previous neuroimaging studies, thus suggesting that structural and functional abnormalities resulting from sleep deprivation were age-dependent (Long & Cheng, 2019; Long, Cheng & Lei, 2020; Richards et al., 2017; Rogasch et al., 2009).

Several limitations in current study need to be mentioned. First, the present study might have limited statistical power due to small sample size. Future researches should involve a larger sample size of participants. Second, the KSQ sleep quality score and ISI score were not recorded after sleep restriction. Thus, we can not investigate the relationship between sleep deprived thalamic alteration and changes of sleep quality. Future studies are warranted to include the sleep quality index in both conditions.

## Conclusions

In conclusion, the current study investigated age-related changes in the thalamic shape and FC variability after three hours of sleep restriction. We found significant local atrophy in the thalamus, and an age × deprivation interaction effect with reduced variability of FC between the left thalamus and the left SPC following sleep restriction only in young adults. These results highlighted the role of the effect of age in studies on sleep deprivation.

##  Supplemental Information

10.7717/peerj.10751/supp-1 Figure S1 Age × deprivation interaction effect using sliding window length of 20 TR Significant agexdeprivation interaction effect (Gaussian Random Field method with *p* < 0.05, voxel *p* < 0.01) on functional connectivity variability of left thalamus (seed) after three hours of sleep restriction. Results were observed under conditions in which the sliding window length was 20 TR. SPC: superior parietal cortex; pre-D: pre-deprivation; post-D: post-deprivation; n.s.: no significance.

10.7717/peerj.10751/supp-2 Figure S2 Age × deprivation interaction effect using sliding window length of 40 TR Significant age × deprivation interaction effect (*p* < 0.01, uncorrected) on functional connectivity variability of left thalamus after three hours of sleep restriction. The results were observed under conditions in which the sliding window length was 40 TR. SPC: superior parietal cortex; pre-D: pre-deprivation; post-D: post-deprivation; n.s.: no significance.

10.7717/peerj.10751/supp-3 File S1 Main effect of deprivation of thalamic shape Th *.nii file can be opened using mricron software. This can be opened using Mricron software (https://www.nitrc.org/projects/mricron).

10.7717/peerj.10751/supp-4 File S2 Age × deprivation interaction effect of left thalamic FC variability Th *.nii file can be opened using mricron software. This can be opened using Mricron software (https://www.nitrc.org/projects/mricron).

## Additional Information and Declarations

### Data Availability

The following information was supplied regarding data availability: Raw data are available in the Supplemental Files.
