# Supplementary figures and images for: Age-related abnormalities of thalamic shape and dynamic functional connectivity after three hours of sleep restriction
Source: PeerJ. 2021 Jan 26;9:e10751. doi: 10.7717/peerj.10751 (PMC7845526; doi:10.7717/peerj.10751)

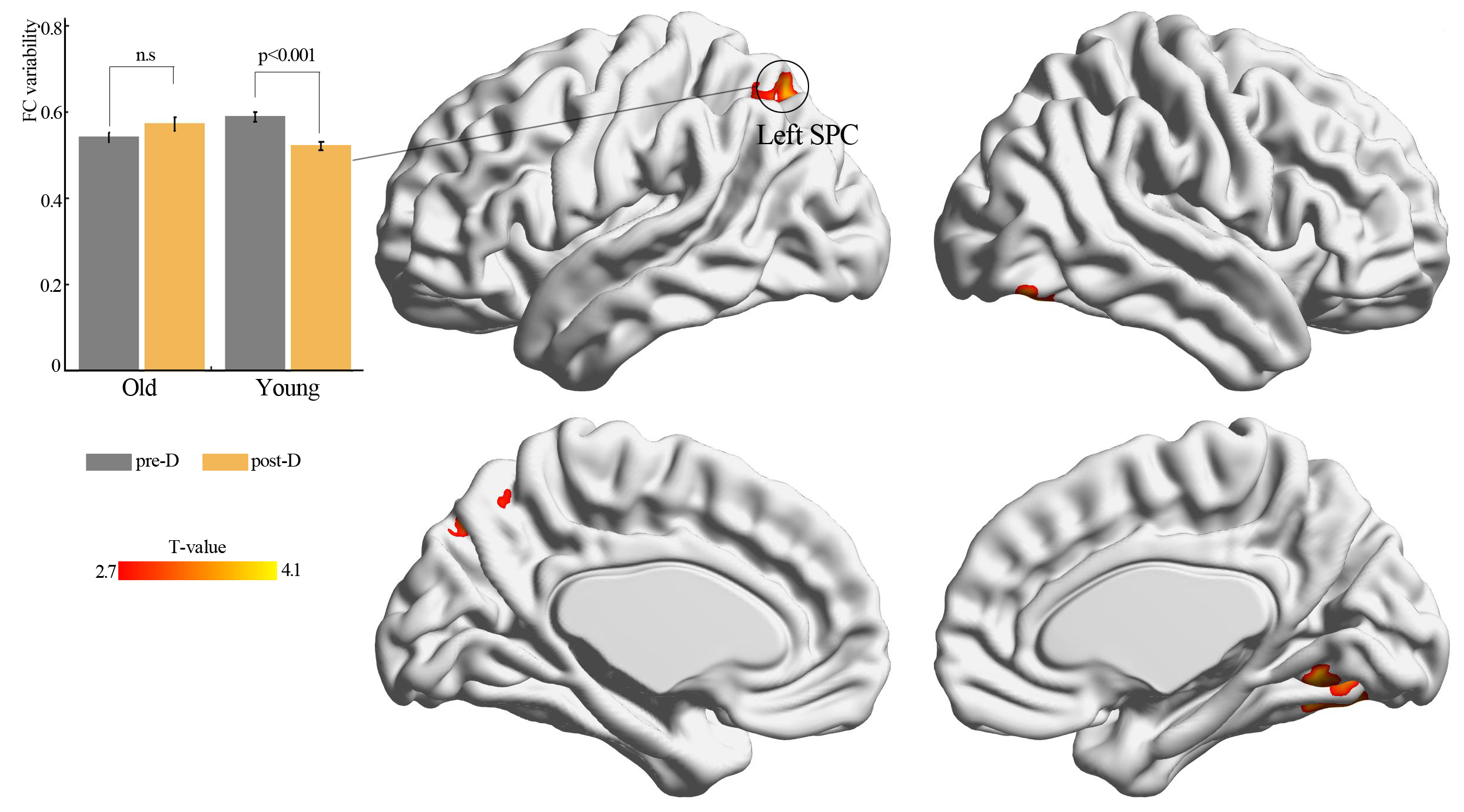

Supplement: Figure S1 — Significant agexdeprivation interaction effect (Gaussian Random Field method with p < 0.05, voxel p < 0.01) on functional connectivity variability of left thalamus (seed) after three hours of sleep restriction. Results were observed under conditions in which the sliding window length was 20 TR. SPC: superior parietal cortex; pre-D: pre-deprivation; post-D: post-deprivation; n.s.: no significance. [file peerj-09-10751-s001.png]

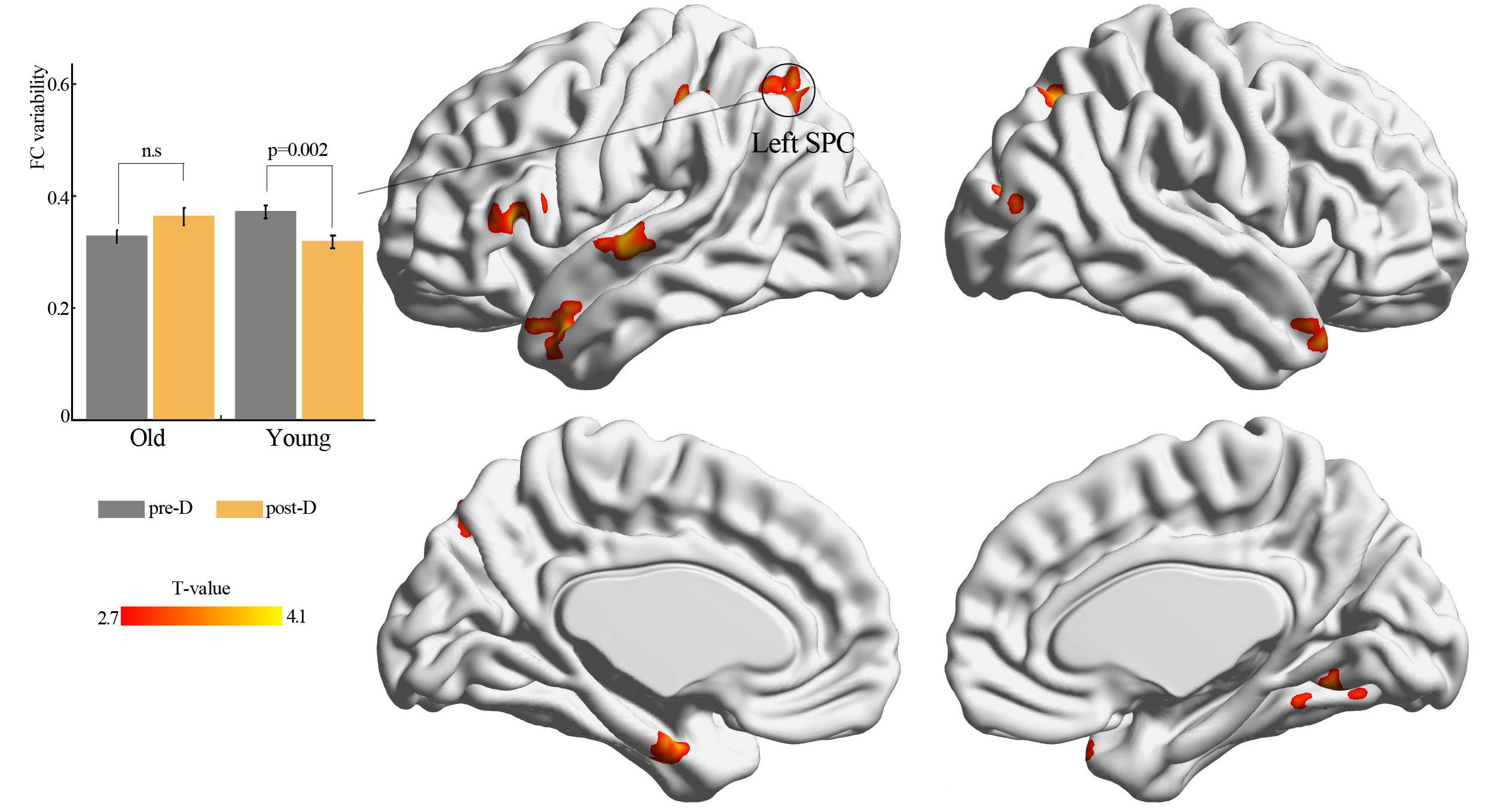

Supplement: Figure S2 — Significant age × deprivation interaction effect (p < 0.01, uncorrected) on functional connectivity variability of left thalamus after three hours of sleep restriction. The results were observed under conditions in which the sliding window length was 40 TR. SPC: superior parietal cortex; pre-D: pre-deprivation; post-D: post-deprivation; n.s.: no significance. [file peerj-09-10751-s002.png]
